# New Paradigms in the Diet and Microbiome Relationship

**DOI:** 10.3390/nu15245035

**Published:** 2023-12-08

**Authors:** Sonia González

**Affiliations:** Department of Functional Biology, University of Oviedo, 33006 Oviedo, Spain; soniagsolares@uniovi.es

Decades of extensive scientific research have led to a consensus on the modulatory effect of diet in shaping the composition and activity of the gut microbiota. The gut (and in particular, the colon) represents the part of the human body most densely populated with micro-organisms (about 95% of the total number living in the human body), comprising bacteria, archaea, viruses and some unicellular eukaryotes that have co-evolved with humans in a commensal manner. This microbiota plays a crucial role in the preservation of the normal physiology of the host [1].

This Special Issue of *Nutrients* collected review and research articles with a common feature, the assessment of the impact of several types of diets and functional foods on the makeup of microbiota structure and activity, and consequently on health status. Research conducted on diets ranging from the Mediterranean to the ketogenic diet, as well as on the creation of vegetable beverages with lactic ferments, illustrates the complexities of this field of study, which is increasing.

Within the conditions closely linked to the composition of the intestinal microbiome are neurological diseases such as epilepsy, Alzheimer’s disease and autism [2,3]. Lim et al., in a comprehensive review, proposed the necessity to further investigate the role of the microbiota as a connector in the protective effects of this kind of diet on health [4]. For this purpose, several recent studies have been compiled in which this restrictive diet was associated with changes in some bacteria of the *Proteobacteria phylum* (*Pseudomonadota*), which are mainly involved in pro-inflammatory responses [5,6]. Even though these data open the way for new research in the field of the gut–brain axis, the authors highlighted the current limitations as well as limitations in reaching future scientific goals, and the possible detrimental health effects of this dietary pattern [7,8,9]. These authors also raised the controversial issue of the use of this ketogenic diet in extreme obese subjects, in whom other pathologies such as diabetes or hypercholesterolemia are usually present, to contribute to weight loss by means of microbiota modulation. Similarly to the above-mentioned studies on neurological disorders, they also focused on a reduction in some bacterial groups such as Proteobacteria against the increase in the phylum Firmicutes, proposing these changes as being useful tools for the management of obesity [10].

Food science has to evolve as society is changing, and in this regard it becomes relevant the review in which Mojikon D et al. argue for the need for a shift in the production of probiotic beverages in line with current lifestyle and consumer preferences [11,12]. In recent years, dairy beverages have been the predominant drinks in the probiotic market [13]. However, the presence of soy-based drinks, lactose intolerance and the rise of veganism have led companies to shift towards the use of plant-based drinks for probiotic purposes [14]. The review provides some reasons why the use of plant-based alternatives may be healthier. It compares the differential aspects between drinks of cereal or fruit and vegetable origin and discusses how these may influence the selection of a particular probiotic. In addition, the mechanisms through which carbohydrates in plant-based beverages can be metabolised by bacteria into metabolites such as short-chain fatty acids with a recognised impact on health are explained in detail. Finally, total soluble solids and sugar consumption are proposed as quality indicators of the fermented product, together with the amount of acid, the pH and the stability at a low temperature.

This Special Issue closes with a research paper by Zapico et al. [15], which shows the impact of implementing an educational program combined with an economic aid in order to promote a healthy Mediterranean-type diet in a group of people at risk of vulnerability. The results presented in this article illustrate the dietary targets in this sample group and how improvements in some dietetic variables, such as the consumption of fruits, vegetables and fish, have an impact on the composition and activity of the gut microbiota and on the presence of depressive symptoms [16].

The determination of potentially carcinogenic compounds derived from food processing and polyphenols with a high level of detail in association with the microbiota is possibly the most innovative aspect of this work [17]. Authors’ reported a decrease in the intake of polycyclic aromatic hydrocarbons (PAHs) and nitrosamines after the intervention.
